# New Bifunctional Bis(azairidacycle) with Axial Chirality via Double Cyclometalation of 2,2′-Bis(aminomethyl)-1,1′-binaphthyl

**DOI:** 10.3390/molecules26041165

**Published:** 2021-02-22

**Authors:** Yasuhiro Sato, Yuichi Kawata, Shungo Yasui, Yoshihito Kayaki, Takao Ikariya

**Affiliations:** 1 Department of Chemical Science and Engineering, School of Materials and Chemical Technology, Tokyo Institute of Technology, 2-12-1-E4-1 O-okayama, Meguro-ku, Tokyo 152-8552, Japan; satouy@fri.go.jp (Y.S.); step_by_step@dk.pdx.ne.jp (Y.K.); yasui0226@gmail.com (S.Y.); 2 Hazardous Materials Laboratory, Research and Development Division, National Research Institute of Fire and Disaster, Jindaiji-higashimachi 4-35-3, Chofu, Tokyo 182-8508, Japan

**Keywords:** bifunctional catalyst, metal-ligand cooperation, dinuclear complex, iridacycle, cyclometalation, asymmetric transfer hydrogenation, cyanation, 1,1-binaphthyl, axial chirality

## Abstract

As a candidate for bifunctional asymmetric catalysts containing a half-sandwich C–N chelating Ir(III) framework (azairidacycle), a dinuclear Ir complex with an axially chiral linkage is newly designed. An expedient synthesis of chiral 2,2′-bis(aminomethyl)-1,1′-binaphthyl (**1**) from 1,1-bi-2-naphthol (BINOL) was accomplished by a three-step process involving nickel-catalyzed cyanation and subsequent reduction with Raney-Ni and KBH_4_. The reaction of (*S*)-**1** with an equimolar amount of [IrCl_2_Cp*]_2_ (Cp* = η^5^–C_5_(CH_3_)_5_) in the presence of sodium acetate in acetonitrile at 80 °C gave a diastereomeric mixture of new dinuclear dichloridodiiridium complexes (**5**) through the double C–H bond cleavage, as confirmed by ^1^H NMR spectroscopy. A loss of the central chirality on the Ir centers of **5** was demonstrated by treatment with KOC(CH_3_)_3_ to generate the corresponding 16e amidoiridium complex **6**. The following hydrogen transfer from 2-propanol to **6** provided diastereomers of hydrido(amine)iridium retaining the bis(azairidacycle) architecture. The dinuclear chlorido(amine)iridium **5** can serve as a catalyst precursor for the asymmetric transfer hydrogenation of acetophenone with a substrate to a catalyst ratio of 200 in the presence of KOC(CH_3_)_3_ in 2-propanol, leading to (*S*)-1-phenylethanol with up to an enantiomeric excess (ee) of 67%.

## 1. Introduction

**Scheme 1:**
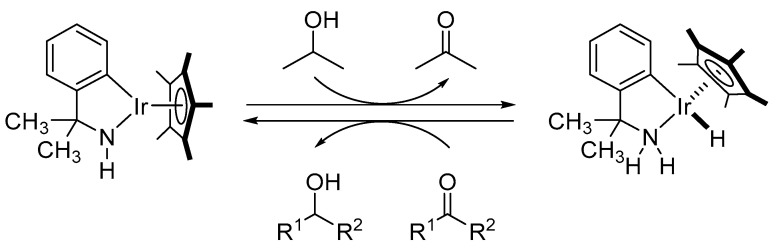
Transfer hydrogenation between alcohols and ketones based on the metal/NH bifunctionality.

The metal/NH bifunctional catalysis has become a pivotal concept for redox transformations based on hydrogen transfer between secondary alcohols and ketones [1,2,3,4,5]. From in-depth studies on original (η^6^-arene)Ru catalysts bearing chiral *N*-sulfonyldiamines (N–N chelate complexes) developed by Noyori and Ikariya [6,7], a fundamental hydrogen delivery process associated with alternating back and forth between 16e amido and 18e hydrido(amine) complexes has been thoroughly realized. We have systematically investigated how the interconversion operates in a range of group 8 and 9 metal complexes with protic amine chelates, and we also investigated how ligand modification, by changing the chelating atom, significantly influences the catalyst performance [2,8,9]. In particular, half-sandwich (η^6^-arene)Ru, Cp*Ir, and Cp*Rh complexes containing a five-membered bifunctional C–N chelate ring, which were synthesized by the orthometalation of protic benzylamine derivatives [10,11,12,13], have been applied to efficient bifunctional catalysts based on the amine/amido functionalities (Scheme 1) [14,15,16,17,18,19,20,21,22,23,24,25,26]. Compared to the prototype N–N chelate Ir complex, remarkable enhancement in the transfer hydrogenation of ketones in 2-propanol [12] and in the aerobic oxidation of alcohols were observed [13]. The rapid hydrogen transfer from alcohols to the amidoiridium and from the hydrido(amine)iridium to ketones also accommodates the dynamic kinetic resolution of racemic secondary alcohols by combination with enzymatic transesterification using *Candida antarctica* lipase B [21]. The unique catalytic function could be enhanced by the high basicity of the amido moiety as well as strong nucleophilicity of the hydrido ligand with the aid of the pronounced σ-donor nature of the coordinating carbon atom.

The asymmetric versions of amidoiridium have been synthesized by the orthometalation of 1-phenyl-1-aminoalkanes with a chiral center at the benzylic position and have provided reasonable enantioselectivities in the asymmetric transfer hydrogenation of acetophenone [25]. The structural studies on the isolable catalytic intermediates suggested that the effective asymmetric induction is intimately bound up with decent diastereomeric control in the formation of hydrido(amine)iridium possessing central chirality at the metal by a one-sided approach of the hydrogen donor, 2-propanol, to amidoiridium. Related cationic Ru, Rh, and Ir complexes bearing cyclometalated C–N chelates were intensively investigated by Pfeffer and coworkers [14,15,16,17,18,19,20] from screening experiments using a variety of chiral benzylic amine ligands, but catalytically active amido and hydrido(amine) complexes have not been characterized.

Separately, it has been reported that dinuclear complexes, in which the mononuclear structure is extended by bridging ligands, exhibit characteristic properties and favorable catalytic performance compared to the parent mononuclear complexes [27,28,29,30,31,32,33,34]. Thanks to the cooperative effect, in which the reaction sites on multiple metals play different roles, and the unique electronic structure of the conjugated bridging ligands, the catalytic activity and enantioselectivity can be improved by multinuclear assembly. In particular, as beneficial applications of optically active dinuclear transition metal complexes with axial chirality introduced into the bridging moiety, Toste et al. have demonstrated the gold-catalyzed dynamic kinetic resolution of propargylic esters [32] and Sasai et al. have demonstrated the vanadium-catalyzed asymmetric oxidative coupling of aromatic compounds [33,34].

**Scheme 2:**
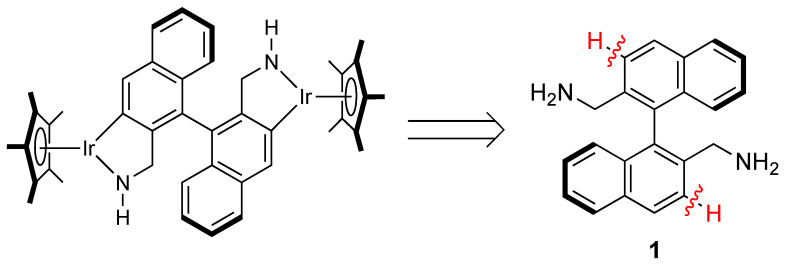
Retrosynthesis of bis(azairidacycle) with an axially chiral binaphthyl linkage.

Encouraged by these works, we focused our interest on a dimetallic compound with a chiral diamido linkage formed by the double orthometalation of benzylic diamine shown in Scheme 2. It is expected that inherent intramolecular steric effects arising from the discrete symmetrical bis(metallacycle) will contribute to enhance the catalytic performance compared with the mononuclear C–N chelate complexes. We report here a synthetic approach toward optically active 2,2′-bis(aminomethyl)-1,1′-binaphthyl (**1**) and its transformation into a new chiral dinuclear azairidacycle. Furthermore, catalytic application to the asymmetric transfer hydrogenation of acetophenone using 2-propanol was also explored.

## 2. Results and Discussion

### 2.1. A Short-Cut Synthesis of 2,2′-Bis(aminomethyl)-1,1′-Binaphthyl

**Scheme 3:**
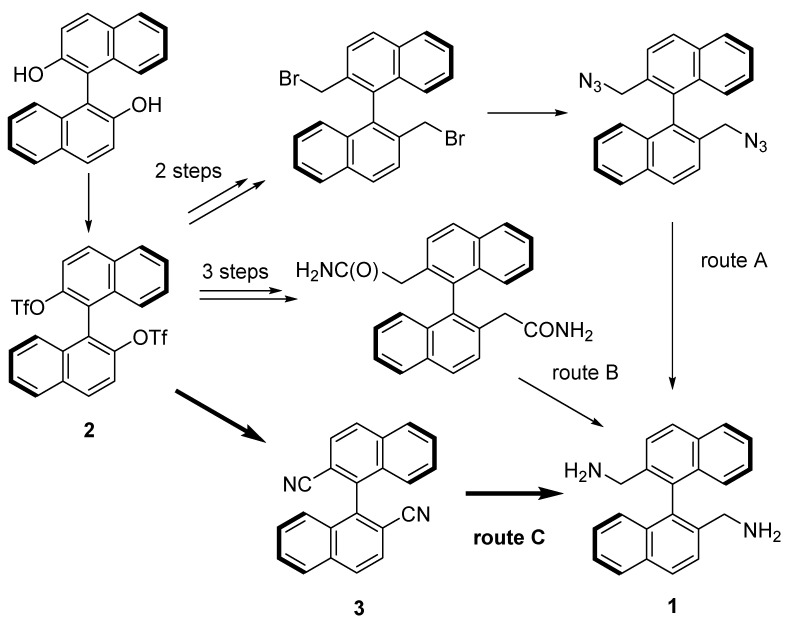
Synthetic routes to 2,2′-bis(aminomethyl)-1,1′-binaphthyl, **1**.

The axially chiral diamine **1** has been synthesized by the reductive transformation of diazo or dicarboxamide compounds as reported independently by Shi [35] and Tomioka [36] (for each route A and B in Scheme 3); however, these precursors are obtained via multistep procedures starting with BINOL. As a simple synthetic strategy enabling C–C bond construction on the 1,1′-binaphthyl scaffold and amine functionalization, we examined a three-step sequence involving the catalytic dicyanation of easily accessible BINOL ditriflate (**2**; >99% enantiomeric excess (ee)) and the following reduction of the C–N triple bonds to the aminomethyl group (route C).

**Scheme 4:**
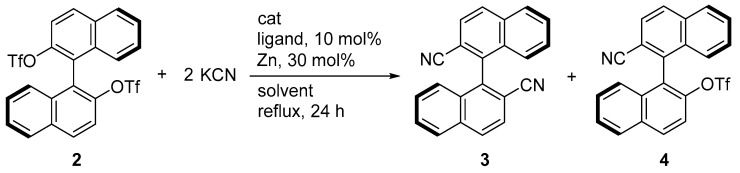
Catalytic cyanation of ditriflate **2**.

**Scheme 5:**
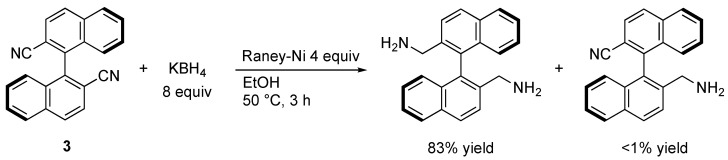
Reduction of **3** with Raney-Ni and KBH_4_.

The cyanation of aryl halides and pseudo-halides with cyanide salts has been disclosed by using Ni or Pd catalysts [37,38,39,40,41,42,43]. To overcome catalyst deactivation by cyanide anion due to its strong coordination ability, some Cu and Zn cocatalysts have been reported to be effective [44,45]. Nevertheless, the employment of **2** as the substrate for dicyanation shown in Scheme 3 remains far less explored, and there is still room for improvement in terms of practicality [46,47]. Although it was reported that a limited amount (10%) of the dinitrile product (**3**) was obtained in a Ni/PPh_3_/Zn three-component catalyst, we found that switching the phosphorus ligand to chelate phosphines resulted in the preferential formation of the dinitrile rather than the mononitrile (**4**) (Scheme 4). Some typical results are summarized in Table 1. When the reaction of (*S*)-**2** with KCN (1.1 equiv) was performed using NiCl_2_(dppe) (10 mol%) with an extra amount (10 mol%) of DPPE (DPPE = 1,2-bis(diphenylphosphino)ethane) in the presence of Zn powder (30 mol %) under reflux conditions for 24 h, a high boiling point solvent, DMF (*N*,*N*-dimethylformamide), was found to be superior to 1,4-dioxane and acetonitrile (entries 1–3). To our delight, (*S*)-**3** was obtained in 87% yield without loss of the optical purity (>99% ee). The use of additional phosphine was advantageous for the cyanation, possibly preventing excessive coordination of the cyanide anion. Other chelate phosphines (entries 4 and 5) and the Pd variant (entry 6) resulted in a decrease in the yield of **3**. The subsequent reduction of **3** was achieved by a combined use of Raney-Ni and KBH_4_ [48], whereas it has been reported that only trace amounts of the desired diamine were produced in some other approaches [47]. After optimization of the reaction conditions, treatment with excess Raney-Ni (4 equiv) and KBH_4_ (8 equiv) in ethanol at 50 °C for 3 h gave (*S*)-**1** in a good yield of 83% (Scheme 5; Figures S4 and S5, In the Supplementary Materials).

### 2.2. Synthesis of Bis(azairidacycle) via Double Cyclometalation

**Scheme 6:**
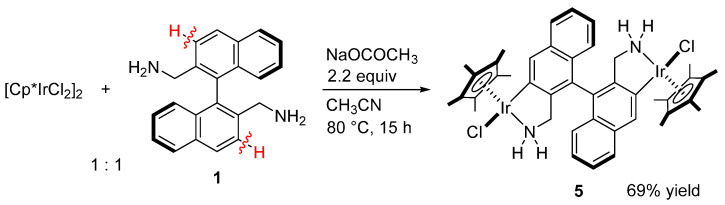
Synthesis of dichloridodiiridium **5** via double orthometalation.

According to our original synthetic procedure for azairidacycles [12], the orthometalation of enantiopure **2** was performed by mixing [Cp*IrCl_2_]_2_ with a 2.2 molar amount of sodium acetate (1.1 equiv/Ir) in acetonitrile at 80 °C for 15 h. After the removal of acetic acid and the remaining salts from the reaction mixture through the work-up process, new dichloridodiiridium (**5**) having an axially chiral 1,1′-binaphthyl linkage was obtained as a yellow powder in 69% yield (Scheme 6). Based on the electrospray ionization mass spectrum (ESI-MS) displaying characteristic ion peaks at *m*/*z* 999.3 and 1001.3 for a fragment of C_42_H_48_N_2_ClIr_2_, a completion of the double cyclometalation was confirmed. The ^1^H NMR spectrum of **5** in CD_2_Cl_2_ exhibited a singlet signal at 1.73 ppm due to the methyl protons of the Cp* ligand along with two minor singlet signals at 1.69 and 1.71 ppm, which suggested the formation of a diastereomeric mixture caused by the chirality at iridium (Figure S6, In the Supplementary Materials).

**Scheme 7:**
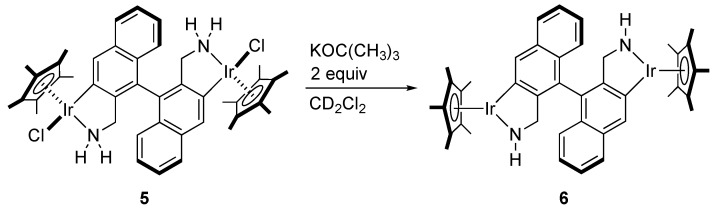
Formation of coordinatively unsaturated amidoiridium complex by dehydrochlorination of **5**.

**Figure 1:**
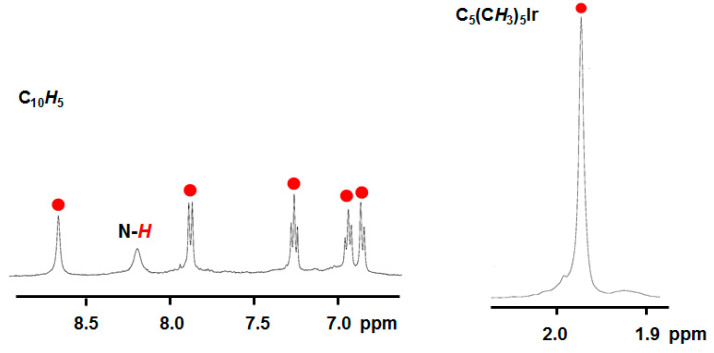
Selected ^1^H NMR spectrum of **6** in CD_2_Cl_2_.

In fact, the subsequent treatment of **5** with 2 equivalents of KOC(CH_3_)_3_ in CD_2_Cl_2_ led to the generation of a coordinatively unsaturated amidoiridium species (**6**) with a color change from orange to dark red purple (Scheme 7), thereby simplifying the spectrum owing to a loss of the central chirality at the iridium atoms. As shown in Figure 1, the signal attributed to the methyl group of Cp* ligand converged to a simple peak at 1.98 ppm. In analogy with the mononuclear C–N chelate complexes, a marked downfield shift trend relative to the NH_2_ signals of amine complexes was observed in the ^1^H NMR spectrum of **6** exhibiting the amido signal at 8.20 ppm. These spectroscopic results corroborate that reactant **5** was comprised of the stereoisomers of chlorido(amine)iridium.

**Scheme 8:**
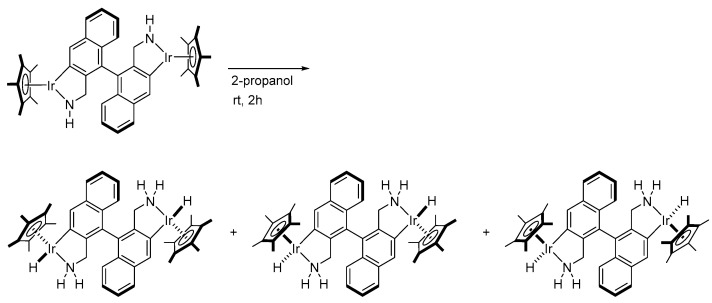
Transformation of **6** into hydrido(amine)iridium complexes.

**Figure 2:**
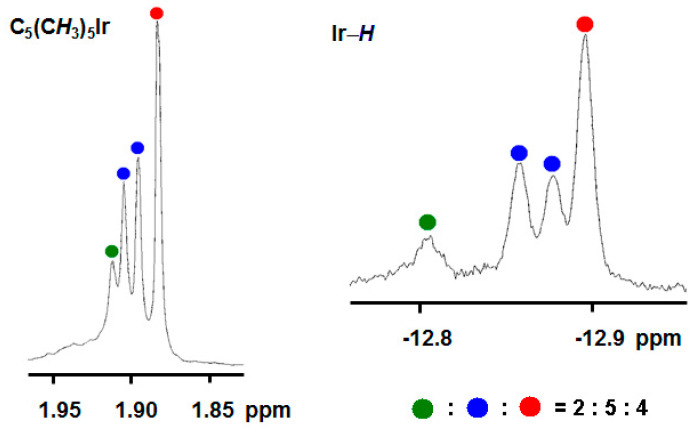
Selected ^1^H NMR spectrum of a streoisomeric mixture of hydridoiridium generated from **6** with 2-propanol.

The 16-electron amidoiridium complex **6** was convertible into the corresponding hydrido(amine)iridium quantitatively in 2-propanol at room temperature for 2 h (Scheme 8), in a similar fashion to the mononuclear complexes [12,25]. After removal of the solvent from the reaction mixture under reduced pressure, the resulting white solid was dissolved in CD_2_Cl_2_, and the ^1^H NMR spectrum was recorded. As shown in Figure 2, four signals in the hydride region at –12.81, –12.86, −12.88, and –12.90 ppm are attributable to formation of three diastereomers involving two homochiral (*R*_Ir_*R*_Ir_ and *S*_Ir_*S*_Ir_ forms) and one hererochiral (an *R*_Ir_*S*_Ir_ form) diiridium centers (Scheme 8). Given the fact that the two signals with identical intensity at –12.86 and –12.88 ppm can be assigned to the latter diastereomer having inequivalent C–N chelating hydridoiridium fragments, the stereoisomers were formed in a ratio of 2:4:5. The ^1^H NMR spectrum also showed singlet signals with the similar intensity ratio at around 1.9 ppm due to the methyl protons of Cp* ligand coordinated to the *C*_2_-symmetric bis(azairidacycle)s and the unsymmetrical stereoisomer. The relative ratios were not altered by lowering the measurement temperature.

### 2.3. Catalytic Application to the Asymmetric Transfer Hydrogenation of Acetophenone in 2-Propanol

**Scheme 9:**
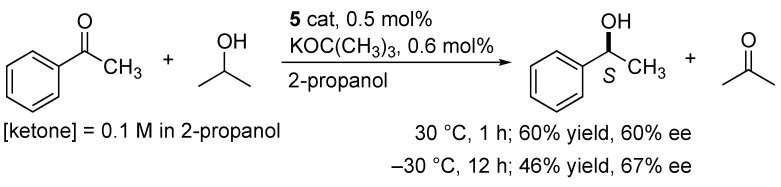
Asymmetric transfer hydrogenation of acetophenone in 2-propanol catalyzed by **5**.

Based on the smooth formation of amidoiridium and hydrido(amine)iridium in the bis(azairidacycle) system, we further explored its catalytic potential for the asymmetric transfer hydrogenation. The reaction of acetophenone in 2-propanol containing the catalyst precursor **5** with a substrate/iridium ratio of 200 at 30 °C proceeded to give (*S*)-1-phenylethanol in 60% yield with a moderate ee of 60% after 1 h (Scheme 9). When the reduction was carried out at −30 °C for 12 h, a slightly higher enantioselectivity (67% ee) was observed, albeit in 46% yield. For the mononuclear C–N chelating ruthenium variants, it has been argued that precise control of the central chirality at the metal of the catalytically active hydrido complexes having three-legged piano stool geometry could be vital for the asymmetric induction through the hydrogen transfer [16]. The above-mentioned formation of minor diastereomers of hydrido(amine)iridium possibly eroded the ee of the chiral secondary alcohol product. The catalytic results indicate that the asymmetric dinuclear complex containing bis(azairidacycle) substructure can behave as a promising bifunctional catalyst for highly efficient transfer hydrogenation, although a redoubled effort to design the chiral ligand structure would be required for dramatic improvement in terms of the discrimination of prochiral face of the ketonic substrates.

## 3. Materials and Methods

### 3.1. General Information

All manipulations of oxygen and moisture-sensitive materials were performed under a purified argon atmosphere using standard Schlenk techniques. Solvents were purchased from Kanto Chemical Co., Inc. (Tokyo, Japan) and dried by refluxing over sodium benzophenone ketyl (THF, 1,4-dioxane, and diethyl ether), P_2_O_5_ (CH_3_CN and CH_2_Cl_2_), or CaH_2_ (2-propanol, pentane), and distilled under argon before use. Ethyl acetate and chloroform-*d*_1_ was used as delivered. Dichloromethane-*d*_2_ was degassed by three freeze–pump–thaw cycles and purified by trap-to-trap distillation after being dried with P_2_O_5_. Acetophenone was purchased from Kanto Chemical Co., Ltd. (Tokyo, Japan), degassed, and stored under argon atmosphere. The other reagents were purchased from Sigma-Aldrich Co. LLC. (St. Louis, MO, USA), Tokyo Chemical Industry Co., Ltd. (Tokyo, Japan), Nacalai Tesque Inc. (Kyoto, Japan) and FUJIFILM Wako Pure Chemical Corporation (Osaka, Japan), and used as delivered. The starting Ir complex, [Cp*IrCl_2_]_2_ [49], and Ni and Pd catalysts such as NiCl_2_ (dppe) [50] were prepared according to the procedures described in the literature with modifications. ^1^H and ^13^C{^1^H} NMR spectra were recorded on a JEOL JNM-LA300 and JNM-ECX400 spectrometers (JEOL Ltd., Tokyo, Japan) at around 25 °C unless otherwise noted. The NMR chemical shifts were referenced to an external tetramethylsilane signal (0.0 ppm) by using the signals of residual proton impurities in the deuterated solvents for ^1^H and ^13^C{^1^H} NMR. ESI-MS was acquired with a JEOL JMS-T100LC spectrometer (JEOL Ltd., Tokyo, Japan). Analytical gas chromatography was performed with a Shimadzu GC-17A gas chromatograph equipped with an INNOWAX capillary column (30 m × 0.25 mm i.d.) purchased from Agilent Technologies (Santa Clara, CA, USA). High performance liquid chromatography (HPLC) analysis was performed using a system comprised of a JASCO column oven: CO-1565, a low-pressure gradient unit: LG-1580-02, a pump: PU-1580, a degasser: DG 1580-53, and a UV/VIS detector: UV-1570. Analytical chiral HPLC was performed on a Chiralcel OD column (4.6 mm × 25 cm) purchased from Daicel Chemical Industries (Osaka, Japan), Ltd. with hexane/2-propanol (95/5) as the eluent where baseline separation was obtained.

### 3.2. Synthesis of 2,2′-Bis(aminomethyl)-1,1′-Binaphthyl

#### 3.2.1. Synthesis of (S)-1,1′-Binaphthalene-2,2′-Bis(trifluoromethanesulfonate) (**2**)

To a solution of (*S*)-1,1′-bi-2-naphthol (2.86 g, 10.0 mmol) in CH_2_Cl_2_ (75 mL) was added pyridine (3.16 g, 40.0 mmol), which was followed by a dropwise addition of a solution of (CF_3_SO_2_)_2_O (6.78 g, 24.0 mmol) in CH_2_Cl_2_ (25 mL) at 0 °C. After stirring the reaction mixture at room temperature for 1 h, an aqueous solution of 3N HCl was added and stirred for 30 min. The mixture was neutralized by a saturated aqueous solution of NaHCO_3_, and the organic layer was separated and washed with water and brine. The organic layer containing the product was dried over MgSO_4_ and concentrated under reduced pressure. Purification by flash chromatography on a silica gel afforded **2** (5.43 g, 99%; Figures S1 and S2, In the Supplementary Materials) as a white powder. ^1^H NMR (399.8 MHz, CDCl_3_, RT): *δ* 7.26 (d, ^3^*J*_HH_ = 9.1 Hz, 2H; C_10_*H*_6_), 7.41 (ddd, ^3^*J*_HH_ = 7.0 Hz, 7.4 Hz, ^4^*J*_HH_ = 1.2 Hz, 2H; C_10_*H*_6_), 7.58 (ddd, ^3^*J*_HH_ = 6.9 Hz, 7.2 Hz, ^4^*J*_HH_ = 1.2 Hz, 2H; C_10_*H*_6_), 7.62 (d, ^3^*J*_HH_ = 9.2 Hz, 2H; C_10_*H*_6_), 8.00 (d, ^3^*J*_HH_ = 8.3 Hz, 2H; C_10_*H*_6_), 8.14 (d, ^3^*J*_HH_ = 9.2 Hz, 2H; C_10_*H*_6_). ^13^C{^1^H} NMR (100.5 MHz, CDCl_3_, RT): *δ* 118.2 (q, ^1^*J*_CF_ = 320 Hz, OSO_2_*C*F_3_), 119.4, 123.5, 126.8, 127.4, 128.0, 128.4, 132.1, 132.4, 133.2, 145.5 (*C*_10_H_6_). ^19^F NMR (376.2 MHz, CDCl_3_, RT): *δ* –74.5 (OSO_2_C*F*_3_).

#### 3.2.2. Catalytic Dicyanation of **2**

The Schlenk tube containing ditriflate **2** (1.65 g, 3.00 mmol), 1,2-bis(diphenylphosphino)ethane (DPPE; 120 mg, 0.30 mmol), [1,2-bis(diphenylphosphino)ethane]dichloridonickel (163 mg, 0.30 mmol), zinc powder (58.9 mg, 0.90 mmol), and potassium cyanide (430 mg, 6.60 mmol) was flushed with Ar and a deoxygenated DMF (3 mL) was added via syringe. The reaction mixture was stirred under reflux conditions for 24 h. Then, the solvent was removed in vacuo, and ethyl acetate (100 mL) was added to the residue. The resulting mixture was washed with H_2_O (100 mL × 2) and brine (10 mL), and then dried over anhydrous MgSO_4_. After evaporation of the organic layer, the resulting material was dissolved in CH_2_Cl_2_, and the solution was filtered through a Florisil column. Subsequent purification by flash chromatography on a silica gel afforded (*S*)-1,1′-binaphthalene-2,2′-dicarbonitrile **3** (799.5 mg, 87%; Figure S3) as white microcrystals. ^1^H NMR (399.8 MHz, CDCl_3_, RT): *δ* 7.16 (dd, ^3^*J*_HH_ = 8.6 Hz, ^4^*J*_HH_ = 0.9 Hz, 2H; C_10_*H*_6_), 7.43 (ddd, ^3^*J*_HH_ = 7.0 Hz, 8.5 Hz, ^4^*J*_HH_ = 1.2 Hz, 2H; C_10_*H*_6_), 7.66 (ddd, ^3^*J*_HH_ = 6.7 Hz, 8.3 Hz, ^4^*J*_HH_ = 0.9 Hz, 2H; C_10_*H*_6_), 7.84 (d, ^3^*J*_HH_ = 8.5 Hz, 2H; C_10_*H*_6_), 8.03 (d, ^3^*J*_HH_ = 8.2 Hz, 2H; C_10_*H*_6_), 8.13 (d, ^3^*J*_HH_ = 8.3 Hz, 2H; C_10_*H*_6_). ^13^C{^1^H} NMR (100.5 MHz, CDCl_3_, RT): *δ* 117.5 (Ar*C*N), 111.5, 126.4, 126.8, 128.5, 128.7, 129.2, 130.3, 131.8, 134.8, 140.5 (*C*_10_H_6_).

#### 3.2.3. Reduction of **3**

Potassium borohydride (1.73 g, 32 mmol), Raney Ni (470 mg), and 10 mL of dry ethanol were placed in a 20 mL Schlenk tube; then, **3** (608 mg, 2.00 mmol) was added while stirring. After stirring vigorously at 50 °C for 3 h, the reaction mixture was filtered through a pad of Celite. The solvent was removed in vacuo, and CH_2_Cl_2_ was added to the residue. The solution was washed with water. After evaporation of the organic layer, the resulting material was dissolved in ethyl acetate. The amine product was extracted by 1N HCl into the aqueous layer, as the HCl salt form. The aqueous layer was neutralized by 1N NaOH and extracted with diethyl ether. The organic layer was dried over anhydrous Na_2_SO_4_. Removal of the solvent under reduced pressure gave the diamine product, **1** (518.2 mg, 83%) as a pale yellow oil. ^1^H NMR (399.8 MHz, CDCl_3_, RT): *δ* 3.48 (d, 2H; C*H*_2_NH_2_), 3.54 (d, 2H; CH_2_N*H*_2_), 7.07 (d, ^3^*J*_HH_ = 8.5 Hz, 2H; C_10_*H*_6_), 7.20 (dd, ^3^*J*_HH_ = 7.0 Hz, 8.0 Hz, 2H; C_10_*H*_6_), 7.41 (dd, ^3^*J*_HH_ = 7.3 Hz, 7.7 Hz, 2H; C_10_*H*_6_), 7.72 (d, ^3^*J*_HH_ = 8.5 Hz, 2H; C_10_*H*_6_), 7.91 (d, ^3^*J*_HH_ = 8.3 Hz, 2H; C_10_*H*_6_), 7.99 (d, ^3^*J*_HH_ = 8.5 Hz, 2H; C_10_*H*_6_). ^13^C{^1^H} NMR (100.5 MHz, CDCl_3_, RT): *δ* 44.5 (*C*H_2_NH_2_), 125.8, 126.1, 126.5, 126.6, 128.2, 128.7, 132.9, 133.2, 133.6, 139.2 (*C*_10_H_6_).

### 3.3. Double Cyclometalation of **1**

To a solution of [Cp*IrCl_2_]_2_ (798 mg, 1.00 mmol) in CH_3_CN (5 mL) was added a solution of **1** (1.0 mmol) in CH_3_CN. After stirring for 5 min, NaOAc (197 mg, 2.4 mmol) was added to the reaction mixture. Then, the reaction mixture was stirred at 80 °C for 15 h. The solvent was removed under reduced pressure. After the reaction mixture was extracted in toluene (10 mL) and filtered through a filter paper, evaporation of the filtrate to dryness gave the product **5** as a yellow powder (831 mg, 0.80 mmol). ^1^H NMR (399.8 MHz, CDCl_3_, RT, the major isomer): *δ* 1.71 (s, 30H; C_5_(C*H*_3_)_5_), 6.95 (m, 2H; C_10_*H*_5_), 6.99 (m, 2H; C_10_*H*_5_), 7.28 (dd, 2H; C_10_*H*_5_), 7.75 (dd, 2H; C_10_*H*_5_), 7.94 (s, 2H; C_10_*H*_5_). MS(ESI) calcd for [C_42_H_48_N_2_ClIr_2_]^+^ [M–Cl]^+^ 1001.28, found 1001.25.

### 3.4. Elimination of HCl from **5** by Treatment of KOC(CH_3_)_3_

A stereoisomeric mixture of **5** (0.29 g, 0.28 mmol) and dry KOC(CH_3_)_3_ (0.09 g, 0.8 mmol) in CH_2_Cl_2_ (3 mL) was stirred at room temperature for 30 min. The reaction mixture was filtered through a pad of Celite under Ar. The solvent was removed under reduced pressure. After the residue was dissolved in diethyl ether, the solution was filtered again through Celite under Ar. Evaporation to dryness of the filtrate gave a dark red-purple powder. The product **6** was dissolved in CD_2_Cl_2_ and analyzed by ^1^H-NMR spectroscopy. ^1^H NMR (399.8 MHz, CD_2_Cl_2_, RT): *δ* 1.94 (br, 4H; C*H*_2_), 1.98 (s, 30H; C_5_(C*H*_3_)_5_), 6.87 (m, 2H; C_10_*H*_5_), 6.95 (m, 2H; C_10_*H*_5_), 7.27 (m, 2H; C_10_*H*_5_), 7.88 (m, 2H; C_10_*H*_5_), 8.22 (br, 4H; N*H*), 8.67 (s, 2H; C_10_*H*_5_).

### 3.5. H NMR Observation of the Generation of Hydrido(Amine)Iridium Species from **6**

Amidoiridium **6** (95.0 mg, 0.1 mmol) was dissolved in 2-propanol (10 mL) and stirred at room temperature for 2 h. After removal of the solvent under reduced pressure, the resulting solid was washed with pentane and dried under vacuum. The product dissolved in CD_2_Cl_2_ was transferred to an NMR tube equipped with a J-Young valve and analyzed by ^1^H NMR spectroscopy at room temperature.

### 3.6. Catalytic Asymmetric Transfer Hydrogenation of Acetophenone In 2-Propanol

A 100-mL Schlenk flask was charged with **5** (25.9 mg, 0.025 mmol), KOC(CH_3_)_3_ (6.7 mg, 0.06 mmol), durene (111.7 g, 0.83 mmol; an internal standard), and 2-propanol (100 mL) under Ar atmosphere. After the addition of acetophenone (1.20 g, 10 mmol), the reaction mixture was stirred at 30 or –30 °C for an appropriate period. The yield of (*S*)-1-phenylethanol was determined by GC analysis, and the optical purity was determined by HPLC analysis.

## 4. Conclusions

In summary, we have developed a synthetic route to the bifunctional asymmetric catalyst based on bis(azairidacycle). The chiral ligand precursor **1** was concisely synthesized from (*S*)-BINOL for a total of 72% yield in three steps including the Ni-catalyzed dicyanation of BINOL ditriflate and the subsequent primary amine formation by reduction using Raney-Ni and KBH_4_. The obtained benzylic diamine could be doubly cyclometalated to give a diastereomeric mixture of chloridoiridium **5**, which was convertible to the coordinatively unsaturated amidoiridium in common with the well-defined mononuclear complexes. The metal–ligand cooperation in the amidoiridium complex allowed the smooth transformation into the corresponding hydrido(amine)iridium in 2-propanol. The promotional role of the azairidacycle was also confirmed in the asymmetric transfer hydrogenation of acetophenone, leading to a reasonable enantioselective.ty. Presumably, exquisite control of the diastereoselectivity during formation of the catalytically active hydrido complexes is needed for further improvement of the asymmetric induction ability. These results will be of assistance in the design of new chiral dinuclear catalyst systems with the metal/NH bifunctionality.

## Figures and Tables

**Table 1:** Catalytic cyanation of **2**.

Entry	Cat	Ligand	Solvent	% Yield ^1^	
				3	4
1	NiCl_2_(dppe)	DPPE	Dioxane	2	n.d. ^2^
2	NiCl_2_(dppe)	DPPE	CH_3_CN	19	17
3	NiCl_2_(dppe)	DPPE	DMF	87	0
4	NiCl_2_(dppp)	DPPP	DMF	57	5
5	NiCl_2_(dppf)	DPPF	DMF	17	n.d. ^2^
6	PdCl_2_(dppe)	DPPE	DMF	59	n.d. ^2^

^1^ Isolated yield. ^2^ Not determined.

## Data Availability

The data presented in this study are available in the Supplementary Materials.

## Supplementary Materials

The following are available online: copies of ^1^H and ^13^C{^1^H} NMR spectra of **1**–**3** and selected ^1^H NMR spectrum of **5**.

## Sample Availability

Samples of the compounds are not available from the authors.
