# Supplementary material for: New Bifunctional Bis(azairidacycle) with Axial Chirality via Double Cyclometalation of 2,2′-Bis(aminomethyl)-1,1′-binaphthyl
Source: Molecules. 2021 Feb 22;26(4):1165. doi: 10.3390/molecules26041165 (PMC7926664; doi:10.3390/molecules26041165)
Supplement: Supplementary file 1 [file molecules-26-01165-s001.pdf]

Supplementary Materials

New Bifunctional Bis(azairidacycle) with Axial Chirality via Double Cyclometalation of 2,2'-Bis(aminomethyl)-1,1'-binaphthyl

Yasuhiro Sato [1,2], Yuichi Kawata[1], Shungo Yasui[1], Yoshihito Kayaki[1,*], and Takao Ikariya[1]

[1] Department of Chemical Science and Engineering, School of Materials and Chemical Technology, Tokyo Institute of Technology, 2-12-1-E4-1 O-okayama, Meguro-ku Tokyo 152-8552, Japan

[2] Hazardous Materials Laboratory, Research and Development Division, National Research Institute of Fire and Disaster, Jindaiji-higashimachi 4-35-3, Chofu, Tokyo 182-8508, Japan

*E-mail: ykayaki@o.cc.titech.ac.jp

Contents

	Page
^{1}H and ^{13}C{^{1}H} NMR Spectra	S2

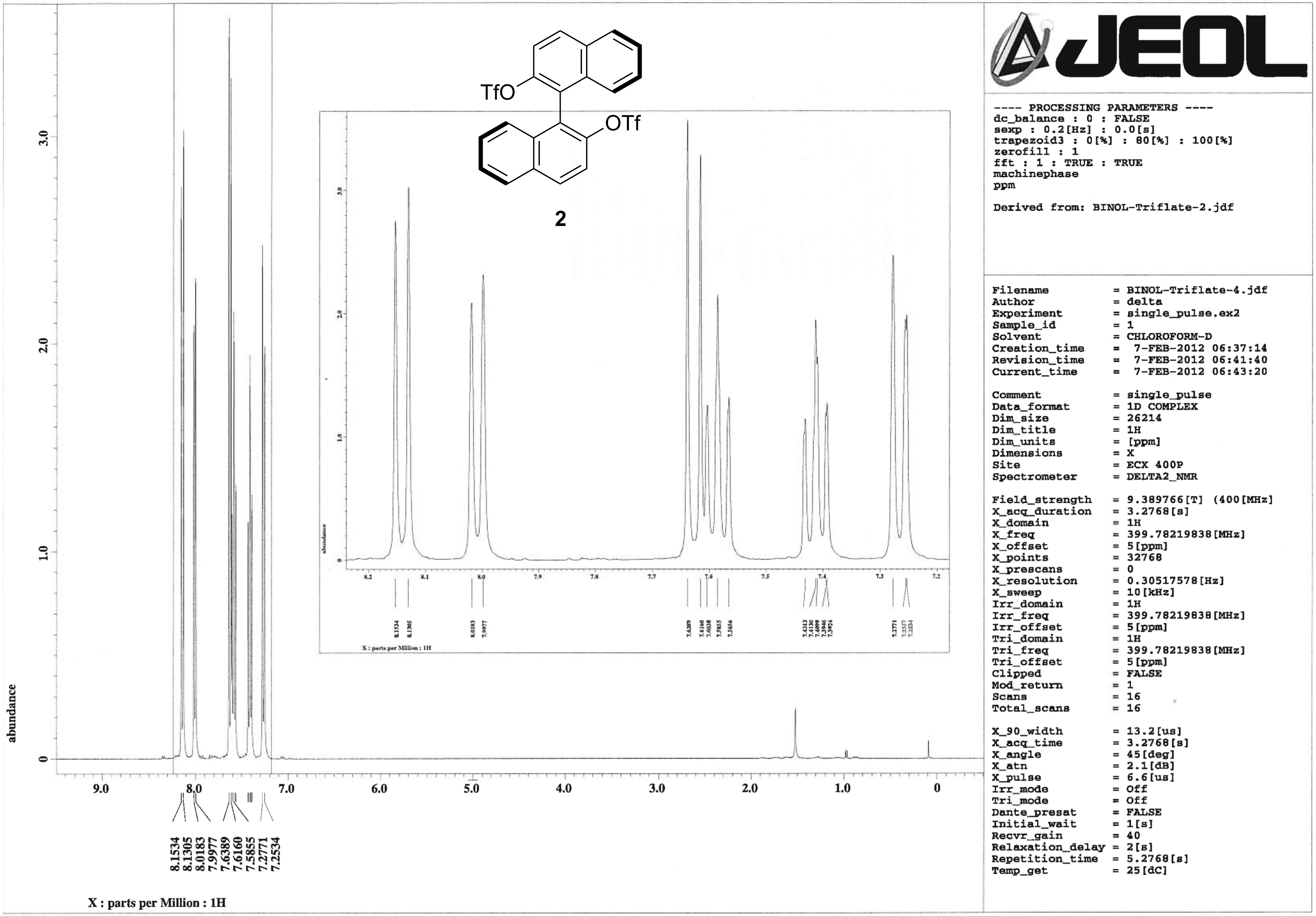

Figure S1. ^{1}H NMR Spectrum of **2**.

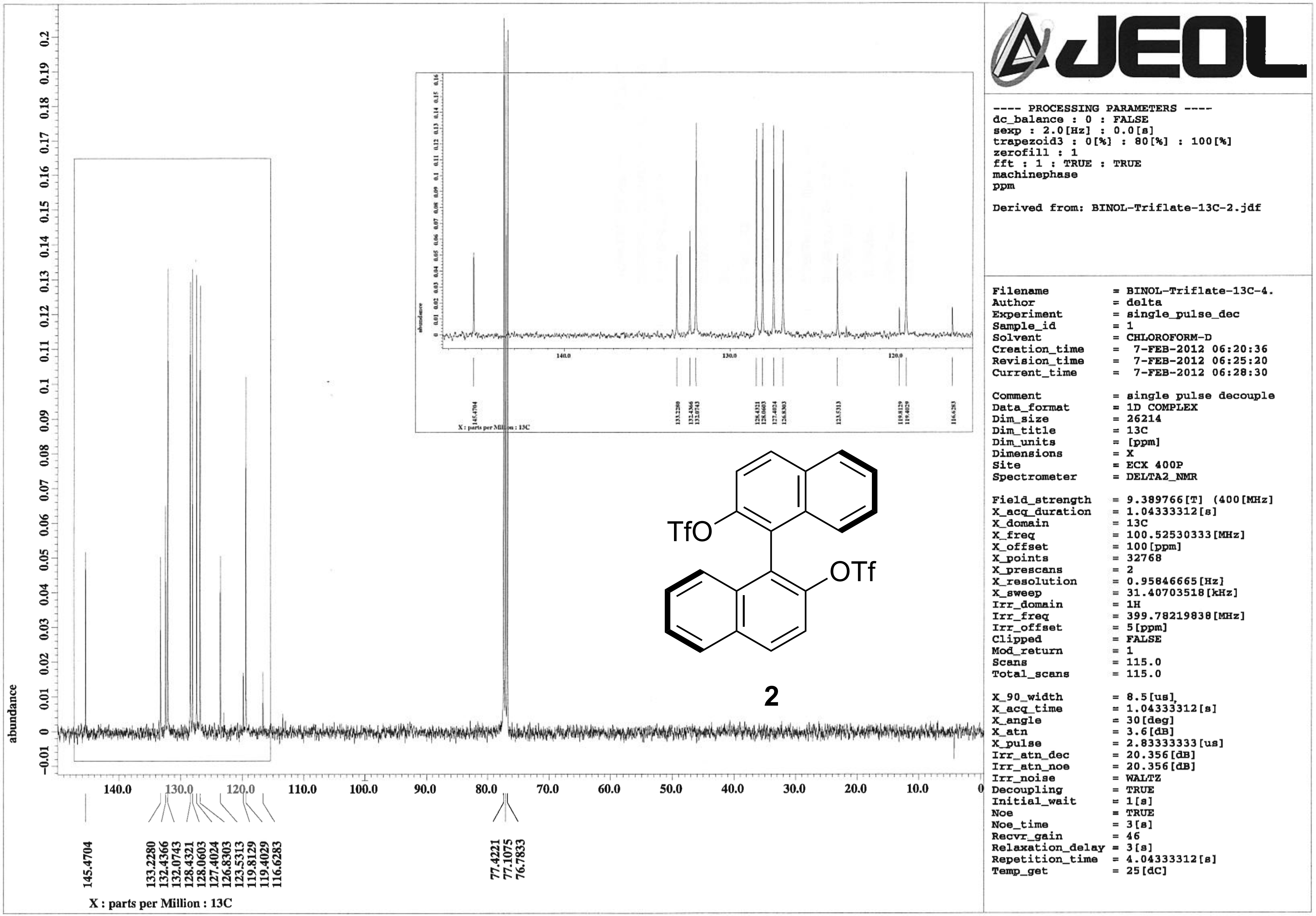

Figure S2. $^{13}C\{^{1}H\}$ NMR Spectrum of **2**.

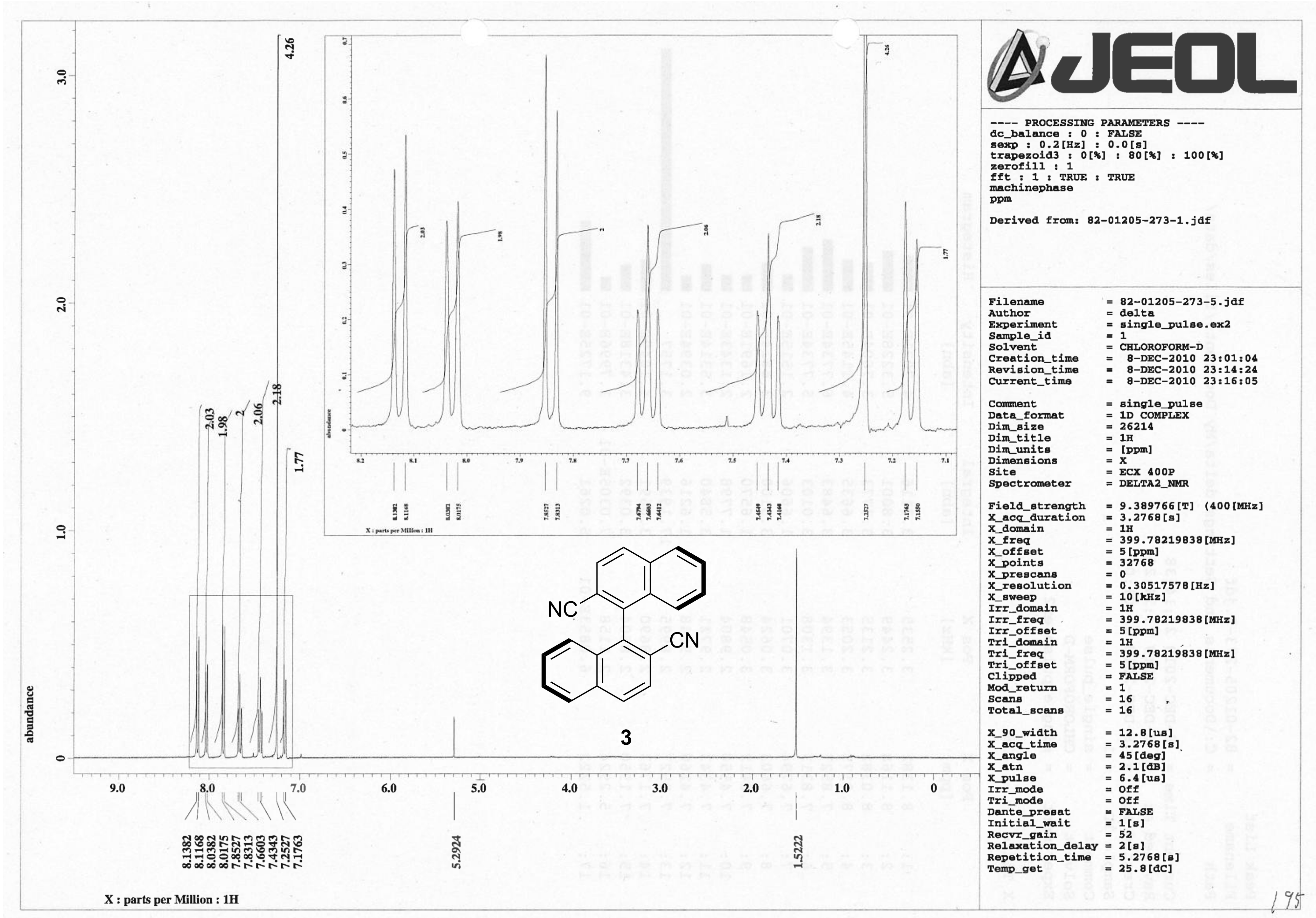

Figure S3. ^{1}H NMR Spectrum of **3**.

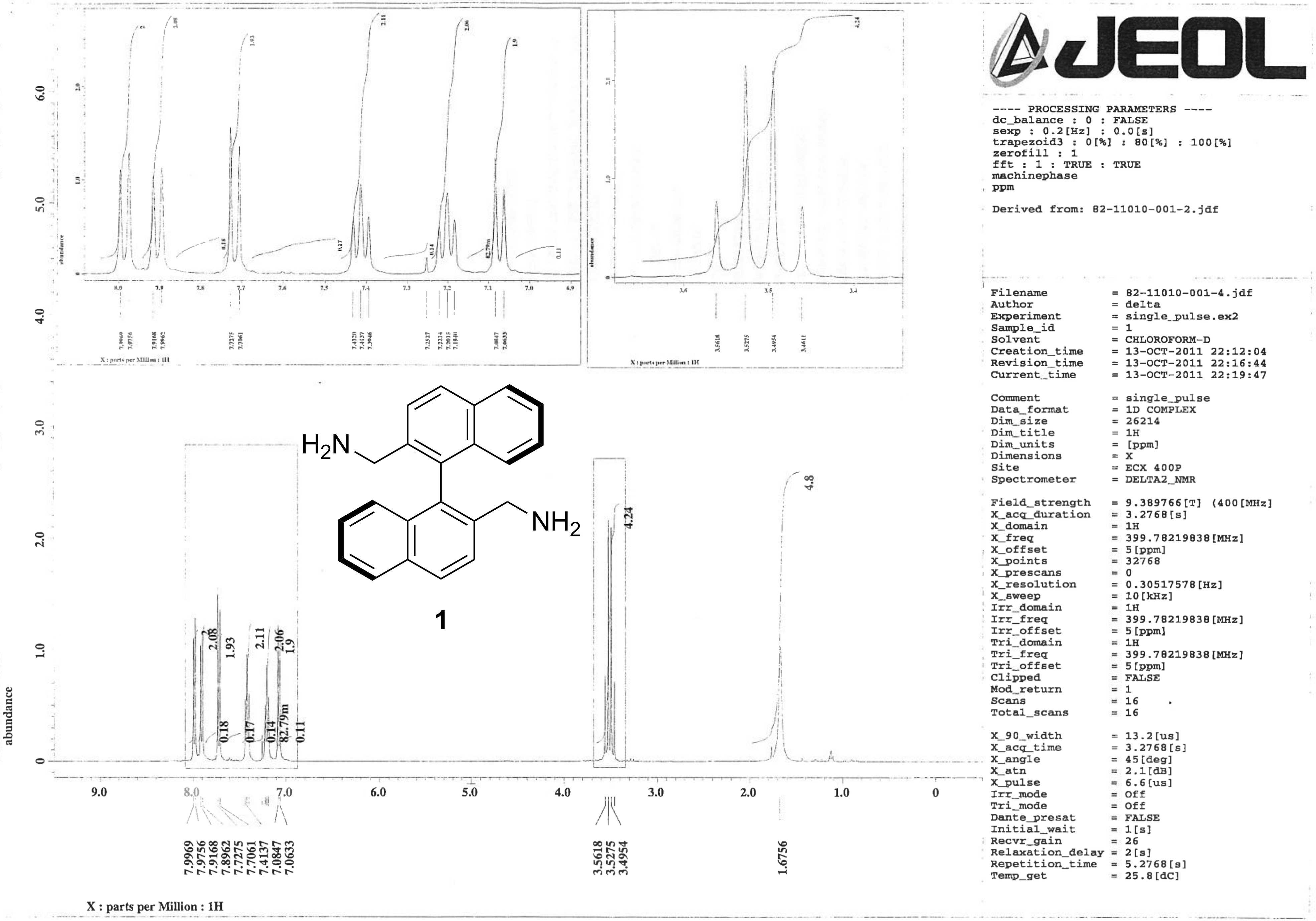

Figure S4. ^{1}H NMR Spectrum of **1**.

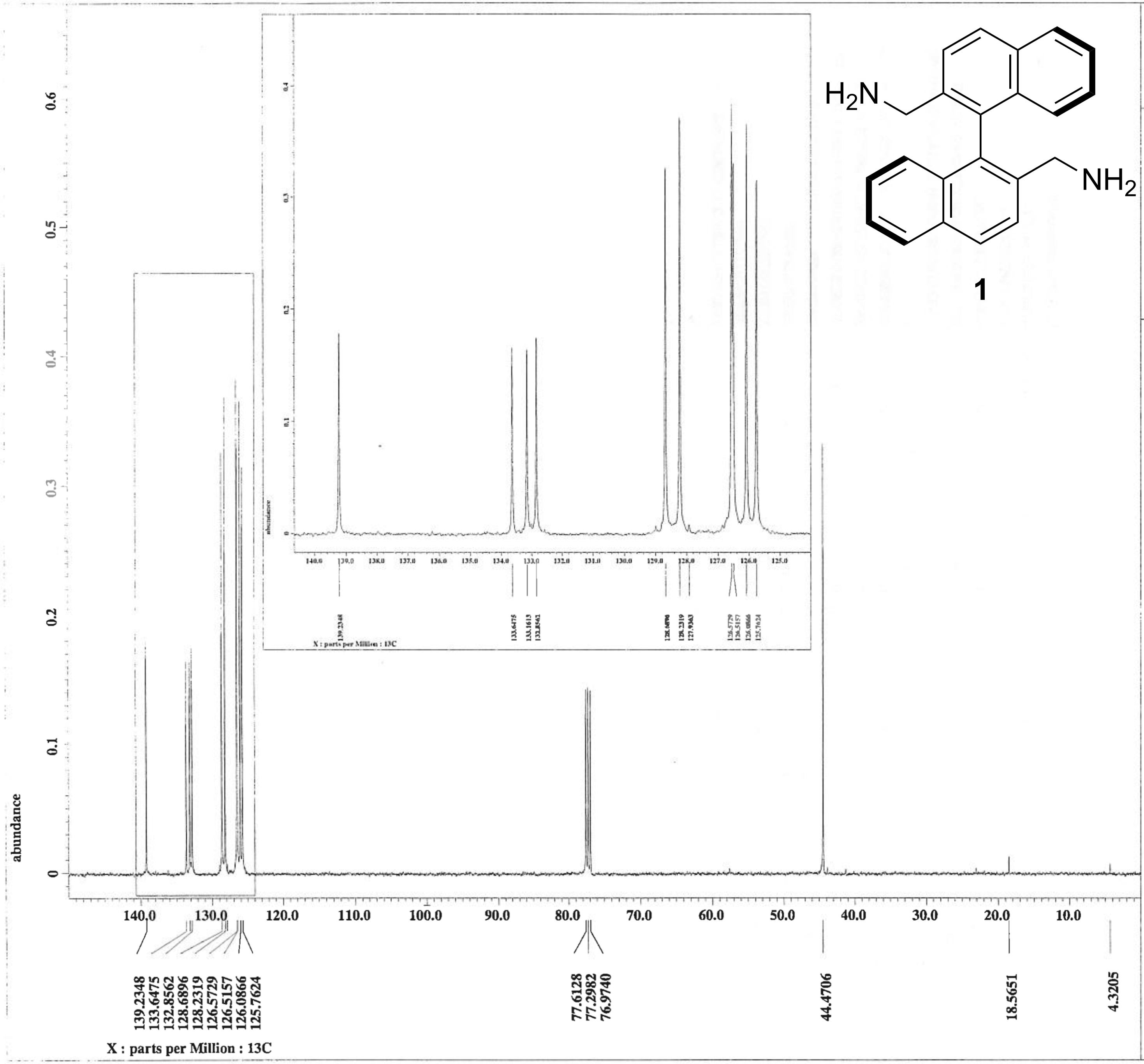

---- PROCESSING PARAMETERS ----
dc_balance : 0 : FALSE
sexp : 2.0[Hz] : 0.0[s]
trapezoid3 : 0[%] : 80[%] : 100[%]
zerofill : 1
fft : 1 : TRUE : TRUE
machinephase
ppm

Derived from: 82-11010-001-13C-1.jdf

Filename	= 82-11010-001-13C-5.jd
Author	= delta
Experiment	= single_pulse_dec
Sample_id	= 1
Solvent	= CHLOROFORM-D
Creation_time	= 13-OCT-2011 22:37:04
Revision_time	= 13-OCT-2011 22:38:01
Current_time	= 13-OCT-2011 22:43:58
Comment	= single pulse decouple
Data_format	= 1D COMPLEX
Dim_size	= 26214
Dim_title	= 13C
Dim_units	= [ppm]
Dimensions	= X
Site	= ECX 400P
Spectrometer	= DELTA2_NMR
Field_strength	= 9.389766[T] (400[MHz]
X_acq_duration	= 1.04333312[s]
X_domain	= 13C
X_freq	= 100.52530333[MHz]
X_offset	= 100[ppm]
X_points	= 32768
X_prescans	= 2
X_resolution	= 0.95846665[Hz]
X_sweep	= 31.40703518[kHz]
Irr_domain	= 1H
Irr_freq	= 399.78219838[MHz]
Irr_offset	= 5[ppm]
Clipped	= FALSE
Mod_return	= 1
Scans	= 274
Total_scans	= 274
X_90_width	= 8.5[us]
X_acq_time	= 1.04333312[s]
X_angle	= 30[deg]
X_atn	= 3.6[dB]
X_pulse	= 2.83333333[us]
Irr_atn_dec	= 20.356[dB]
Irr_atn_noe	= 20.356[dB]
Irr_noise	= WALTZ
Decoupling	= TRUE
Initial_wait	= 1[s]
Noe	= TRUE
Noe_time	= 3[s]
Recvr_gain	= 48
Relaxation_delay	= 3[s]
Repetition_time	= 4.04333312[s]
Temp_get	= 25.9[dC]

Figure S5. $^{13}C\{^{1}H\}$ NMR Spectrum of **1**.

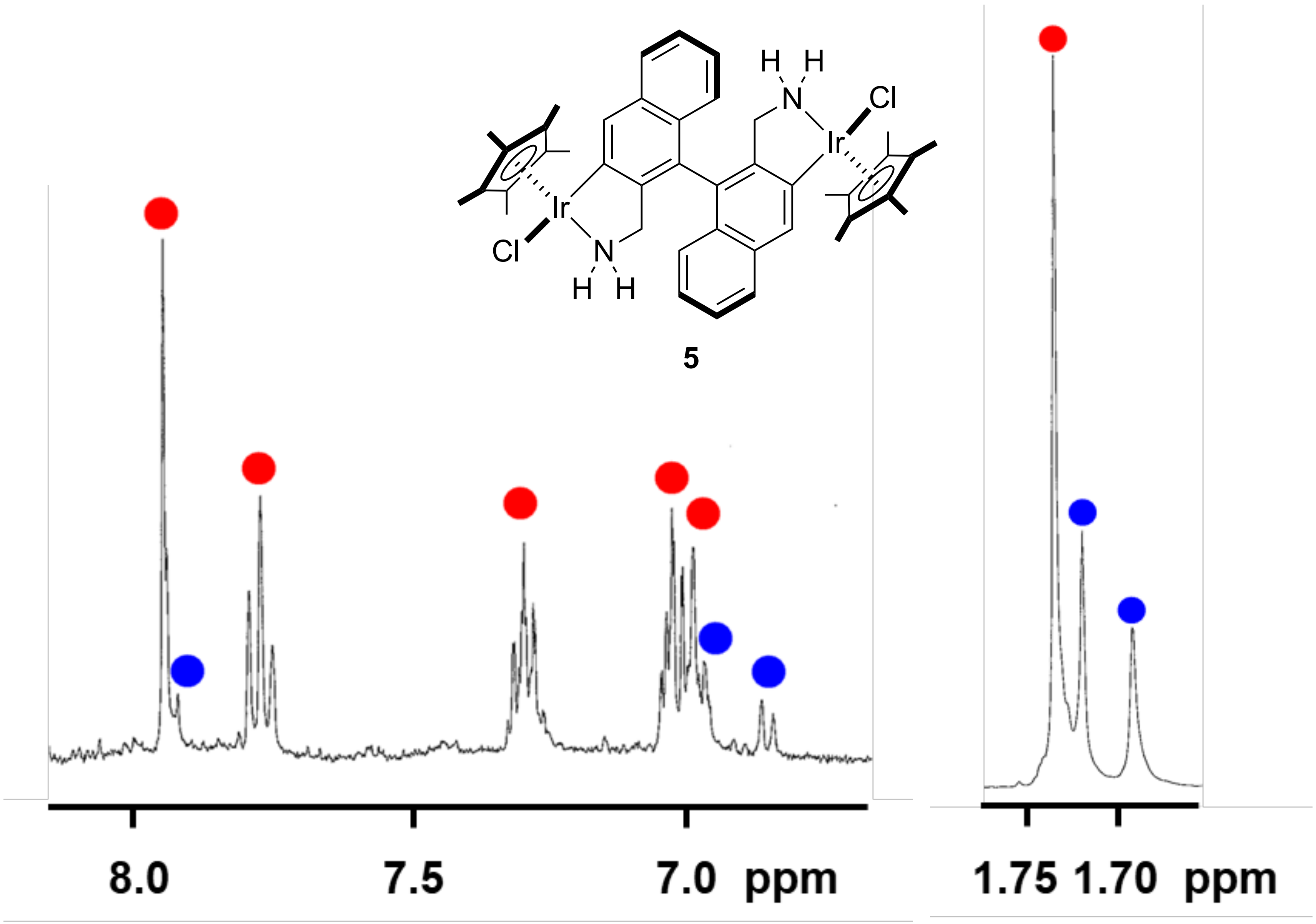

Figure S6. Selected ^{1}H NMR Spectrum of **6**.
